# Immune Activation, Exhaustion and Senescence Profiles as Possible Predictors of Cancer in Liver Transplanted Patients

**DOI:** 10.3389/fonc.2022.899170

**Published:** 2022-06-13

**Authors:** Maria Raffaella Petrara, Sarah Shalaby, Elena Ruffoni, Martina Taborelli, Francesco Carmona, Silvia Giunco, Paola Del Bianco, Pierluca Piselli, Diego Serraino, Umberto Cillo, Riccardo Dolcetti, Patrizia Burra, Anita De Rossi

**Affiliations:** ^1^ Oncology and Immunology Section, Department of Surgery, Oncology and Gastroenterology, University of Padova, Padova, Italy; ^2^ Multivisceral Transplant Unit, Department of Surgery, Oncology and Gastroenterology, Padova University Hospital, Padova, Italy; ^3^ Immunology and Diagnostic Molecular Oncology Unit, Veneto Institute of Oncology IOV-IRCCS, Padova, Italy; ^4^ Cancer Epidemiology Unit, Centro di Riferimento Oncologico di Aviano (CRO) IRCCS, Aviano, Italy; ^5^ Clinical Research Unit, Veneto Institute of Oncology IOV-IRCCS, Padova, Italy; ^6^ Clinical Epidemiology Unit, Istituto Nazionale per le Malattie Infettive (INMI) “L. Spallanzani” IRCCS, Rome, Italy; ^7^ Hepatobiliary Surgery and Liver Transplantation Unit, Department of Surgery, Oncology, and Gastroenterology, Padova University Hospital, Padova, Italy; ^8^ Sir Peter MacCallum Department of Oncology, The University of Melbourne, Melbourne, VIC, Australia; ^9^ Department of Microbiology and Immunology, The University of Melbourne, Melbourne, VIC, Australia

**Keywords:** immune activation, immune senescence, post-transplant malignancy, hepatocellular carcinoma, biological predictors

## Abstract

Liver transplanted (LT) patients for hepatocellular carcinoma (LT-HCC) or for other causes (LT-no-HCC) may develop post-transplantation malignancies. Although immune activation and senescence are frequently implicated in cancer development, no data is available on their possible role as biomarkers predictive of tumor onset in this setting. A total of 116 patients were investigated: the 45 LT-HCC patients were older than the 71 LT-non-HCC (p=0.011), but comparable for sex, HCV, HBV infection and immunosuppressive treatment. At baseline, the numbers of activated and senescent-like circulating cells were significantly higher in LT-HCC patients than in LT-no-HCC ones. After a median follow-up of 26.8 months, 6 post-transplant malignancies (PTM) occurred: 4 in LT-HCC (8.9%) and 2 in LT-no-HCC (2.8%) patients. Overall, subjects with high percentages of activated and exhausted T and B cells at baseline were at higher risk of PTM. Notably, within the LT-HCC group, a higher percentage of senescence-like T cells was also associated with cancer development. Moreover, patients with PTM had higher telomere erosion and higher levels of circulating PAMPs (16S rDNA) and DAMPs (mtDNA) when compared with matched patients without PTM. Overall, these findings suggest that immune activation and exhaustion may be useful to predict the risk of PTM occurrence, regardless of the cause of transplantation. In LT-HCC, T-cell senescence represents an additional risk factor for tumor onset.

## Introduction

Liver transplantation is the treatment of choice for patients with end-stage liver disease, acute liver failure or hepatocellular carcinoma (HCC). In Italy, a total of 12,519 liver transplants were performed between 2010 and 2020 (1). The lifelong use of immunosuppressive anti-rejection treatment substantially increases the risk of post-transplant malignancies (PTM). Although advances in immunosuppressive therapy have contributed to improve patient and graft survival, cancers represent a major adverse outcome in liver transplanted (LT) patients, including a negative impact on morbidity and mortality. Furthermore, PTM are a leading cause of death in LT recipients (2–5).

HCC is the most common type of liver cancer, accounting for 75%–85% of cases (6, 7), and is characterized by rapid progression, frequent metastasis, late diagnosis, high post-operative recurrence and poor prognosis (6). It has been reported that liver transplanted patients for HCC (LT-HCC) tended to be at higher risk of developing *de novo* malignancies (DNMs) compared to liver transplanted patients for other causes (LT-no-HCC) (7). Recently, an earlier onset of solid DNMs in LT-HCC compared to LT-no-HCC has been documented (8).

Chronic inflammation is a well-known factor promoting cancerogenesis, including hepatocarcinogenesis, a multistep process induced by exposure to agents triggering insults in the liver (e.g., chronic viral hepatitis infection, alcohol use) followed by chronic local inflammation (9–11). After transplantation, events such as the ischaemia-reperfusion injury and/or immunosuppressive treatments also induce an inflammatory status through the release of pathogen-associated molecular patterns (PAMPs), and damage-associated molecular patterns (DAMPs) into circulation. The subsequent induction of pro-inflammatory cytokines leads to the activation of immune system, which in turn contributes to maintain the inflammatory environment, a pivotal step for tumorigenesis (12–14).

Senescence may also play an important role in the carcinogenesis process; indeed, senescent cells secrete pro-inflammatory cytokines and growth factors, known as the senescence-associated secretory phenotype (SASP), which have been implicated in both aging and cancer development (15, 16). In a previous study, we found that immune senescence biomarkers were significantly higher in elderly colon cancer patients than in age-matched controls; in particular, cancer patients exhibited higher percentages of senescent CD8^+^ T cells. Consistently, lower thymic output and shorter telomeres were associated with a higher risk of developing cancer in the same cohort (17). Furthermore, cancer patients with high percentage of senescent and activated CD8^+^ T cells had a worse disease outcome (18).

Considering the possible clinical relevance of the availability of suitable biomarkers for the prediction of tumor onset in LT patients, in the present study we have characterized immune activation, exhaustion, and senescence markers in the blood of a prospective series of LT patients with the final goal to assess their possible usefulness in the definition of the risk of PTM development.

## Materials and Methods

### Study Population and Sampling

A total of 116 LT patients have been prospectively enrolled between October 2016 and February 2021 at the Multivisceral Transplant Unit, Padova University-Hospital. Forty-five patients were LT-HCC and 71 LT-no-HCC (i.e. decompensated cirrhosis, cholestatic liver disease, acute liver failure). Patients’ clinical information were recorded and included personal details (age at transplant, sex), transplant details (date of liver transplantation, underlying disease, Model for End-Stage Liver Disease (MELD) score and alpha-fetoprotein levels at liver transplantation) and post-transplantation immunosuppressive schedule, considering modifications during follow-up. For LT-HCC patients, tumor characteristics were assessed through explant pathology evaluation for the following characteristics: number of HCC nodules, maximum nodular diameter, tumor differentiation and presence of macro/micro-vascular invasion. Vital tumor volume was quantified according to the following equation: “Tumor volume mm^3^ = 4/3 x 3.14 x (radius of the tumor nodule in mm^3^)”. Total tumor volume (TTV) was calculated as the sum of the tumor volume of each nodule. LT-HCC patients were followed for HCC recurrence through CT-scan or MRI every 3 months during the first year, and every 6 months thereafter, according to the European Association for the Study of the Liver/European Organization for Research and Treatment of Cancer (EASL-EORTC) clinical practice guidelines for the management of hepatocellular cancer (19). As they were candidates for LT, patients were not treated with systemic anticancer drugs and underwent locoregional bridge treatments.

After a median follow-up of 26.8 months following liver transplantation, 6 patients developed a PTM (LT-PTM): 5 were DNMs and 1 was an HCC recurrence. DNMs were defined as neoplasms developing after transplantation in patients negative at pre-transplant screening for the HCC, or related pre-malignant lesions/conditions. DNMs were coded according to the International Classification of Diseases and Related Health Problems, 10th revision (ICD-10). Diagnosis of DNMs was established by histology on biopsies or surgical specimens of the tumor. Date of biopsy or surgical procedure was designated as the date of cancer diagnosis. All patients underwent a full pre-transplant screening aimed at excluding any type of cancer other than HCC, or related premalignant lesions/conditions, which was negative in all patients. Moreover, to avoid any accidental missing pre-LT diagnosis, lesions that appeared within 6 months after transplantation were excluded from the definition of DNM. In our cohort, the 5 DNMs were: infiltrating ductal carcinoma of the breast, ovarian cancer, larynx carcinoma, basal cell carcinoma of the external auditory canal, and prostate cancer. Among the 110 patients without PTM (LT-no-PTM), 26 subjects were selected as a matched (m)LT-no-PTM control group according to the following inclusion criteria: age, sex difference, immunosuppressive combined strategies, and a median time interval from baseline to follow-up. This study was approved by the Ethical Committee of Padova University Hospital (Prot. 4231/AO/17).

### Sample Collection

Blood samples were collected in EDTA-containing tubes at baseline (at liver transplantation) and at follow-up (6, 12 months and every year after transplantation and/or at cancer onset). From samples at baseline and at closest time to the tumor onset in LT-PTM patients, or with a median time interval similar for mLT-no-PTM patients, peripheral blood mononuclear cells (PBMC) were isolated by centrifugation on a Ficoll-Paque (Pharmacia, Uppsala, Sweden) gradient. PBMC were cryopreserved in liquid nitrogen, and plasma samples at - 80°C, until use.

### Flow Cytometry

**Figure 1:**
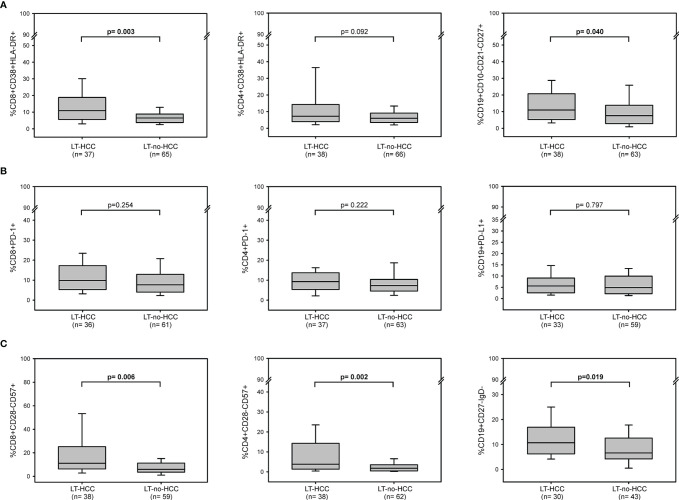
Baseline immune activation, exhaustion and senescence profiles in LT-HCC and LT-no-HCC patients. Percentages of **(A)** activated CD8^+^ (CD3^+^CD8^+^CD38^+^HLA-DR^+^), CD4^+^ (CD3^+^CD4^+^CD38^+^HLA-DR^+^) and memory B (CD19^+^CD10^-^CD21^-^CD27^+^) cells; **(B)** exhausted CD8^+^ (CD3^+^CD8^+^PD-1^+^), CD4^+^ (CD3^+^CD4^+^PD-1^+^) and B (CD19^+^PD-L1^+^) cells, **(C)** senescent-like CD8^+^ (CD3^+^CD8^+^CD28^-^CD57^+^), CD4^+^ (CD3^+^CD4^+^CD28^-^CD57^+^), and B (CD19^+^CD27^-^IgD^-^) cells in LT-HCC and LT-no-HCC patients. All p-values were adjusted by age.

Immunophenotyping was performed on cryopreserved PBMC. Cells were thawed, washed, stained for 20 min in the dark with the Live/Dead Fixable Near-IR Dead Cell Stain Kit (Life Technologies, Carlsbad, California, USA) and the following labelled monoclonal antibodies (mAbs): anti-CD3 [fluorescein isothiocyanate (FITC)], anti-CD4 [peridinin chlorophyll protein (PerCP)], anti-CD38 [phycoerythrin (PE)], anti-HLA-DR [allophycocyanin (APC)], anti-CD279 (programmed cell death 1, PD-1) [PE-Cy7], anti-CD57 [PE], anti-CD21 [BV421], anti-CD27 [PE-Cy7], anti-IgD [PE], anti-CD274 (programmed cell death ligand 1, PD-L1) [BV421] (Becton-Dickinson, San Diego, California, USA); anti-CD8 [VioGreen], anti-CD28 [APC], anti-CD19 [VioBright515], anti-CD10 [APC] (Miltenyi Biotec, Auburn, California USA). Cells were then washed and resuspended in phosphate-buffered saline supplemented with 1% paraformaldehyde. All samples were analyzed using LSRII Flow cytometer (Becton-Dickinson). A total of 50000 events were collected in the lymphocyte gate using morphological parameters (forward and side-scatter). Data were processed with FACSDiva Software (Becton-Dickinson) and analyzed using Kaluza Analyzing Software v.1.2 (Beckman Coulter) **(** **Figure 1 supplementary** **).**

### Determination of Telomere Length

DNA was extracted from PBMC, using QIAmp DNA Blood Mini Kit (Qiagen, Hilden, Germany) according to the manufacturer’s instructions. Relative telomere length (RTL) was determined by monochrome quantitative multiplex real-time PCR, as previously described (20, 21).

### Quantification of PAMPs and DAMPs

DNA extraction from 200 µl of plasma was performed using QIAamp DNA Mini Kit (QIAGEN, Hilden, Germany), and eluted in 50 µl of AE Buffer. A quantitative method based on real-time PCR assay was performed to quantify plasma levels of 16S rDNA with primer pair (forward 5’-AGTTTGATCCTGGCTCAG-3’ and reverse 5’-GWATTACCGCGGCKGCTG-3’) and probe (5’-FAM-GCTGCCTCCCGTAGGAGT-BHQ-3’), as previously described (22, 23). Results were expressed as 16S rDNA copies/μl plasma. Plasma levels of mitochondrial DNA (mtDNA) were quantified by real-time PCR assay with primer pair (forward 5’-AGGACAAGAGAAATAAGGCC-3’ and reverse 5’-TAAGAAGAGGAATTGAACCTCTGACTGTAA-3’) and probe (5’-FAM-TTCACAAAGCGCCTTCCCCCGTAAATGA-BHQ-3’), as previously described (23, 24). Results were expressed as mtDNA copies/μl plasma.

### Statistical Analysis

Continuous variables were summarized using median and interquartile range (IQR), categorical variables as frequencies and percentages. The median follow-up time was based on the reverse Kaplan-Meier estimator. Clinical characteristics at baseline were compared between LT-HCC and LT-no-HCC patients using the Kruskal-Wallis test, or Fisher exact test as appropriate. A Mann-Whitney test, adjusted for age, was used to address comparisons of each immunological parameter distribution between groups of interest. The impact of the immunological parameters on the probability of experiencing HCC or PTM was estimated in univariate logistic regression models fitted by penalized maximum likelihood to address low frequency data. Each covariate was considered as a categorical variable according to high and low levels. Optimal cut-points were selected using a criterion based on maximizing the Youden index, being the summary measure of the ROC curve, over all possible cut-points. The odds ratios (ORs) were reported with their 95% confidence interval (CI). ORs were also adjusted for age as continuous variable. Subgroup analyses were performed with explorative intent. All statistical tests were two-sided and a p value <0.05 was considered statistically significant. Statistical analyses were performed using the RStudio (RStudio: Integrated Development for R. RStudio Inc., Boston, MA, US).

## Results

### Patients’ Characteristics

The patients’ characteristics are summarized in **Table 1**. Overall, 69% of LT patients were male. Median age was 56.0 years (IQR 46.8-62.0); LT-HCC were significantly older than LT-no-HCC patients: 60.0 years (52.0-63.0) *vs* 53.0 years (45.5-61.0), p=0.011.

**Table 1:** Characteristics of LT-HCC and LT-no-HCC patients.

		Total Patients	LT-HCC Patients	LT-no-HCC Patients	p-value
	N	116	45	71	
**Age (years)**	Median (IQR)	56.0 (46.8-62.0)	60.0 (52.0-63.0)	53.0 (45.5-61.0)	**0.011**
**Sex**	F	36 (31.0%)	10 (22.2%)	26 (36.6%)	0.102
	M	80 (69.0%)	35 (77.8%)	45 (63.4%)	
**HCV**	No	98 (84.5%)	36 (80.0%)	62 (87.3%)	0.288
	Yes	18 (15.5%)	9 (20.0%)	9 (12.7%)	
**HBV**	No	90 (77.6%)	33 (73.3%)	57 (80.3%)	0.493
	Yes	13 (11.2%)	5 (11.1%)	8 (11.3%)	
	Yes + HDV	13 (11.2%)	7 (15.6%)	6 (8.5%)	
**Nodules n.**	Median (IQR)		1 (1- 4)		
**Maximum size**	Median (IQR)		1.70 (0.80-3.00)		
**TTV (cm^3^)**	Median (IQR)		4.19 (0.27-25.11)		
**Vascular invasion**	Macro		3 (7.1%)		
	Micro		6 (14.3%)		
	No		33 (78.6%)		
**Grading**	1		8 (21.6%)		
	2		22 (59.5%)		
	3		7 (18.9%)		
**Follow-up (months)**	Median (IQR)	26.84 (10.22-42.64)	21.52 (7.85-37.91)	29.50 (13.50-43.17)	0.220
**PTM**	No	110 (94.8%)	41 (91.1%)	69 (97.2%)	0.150
	Yes	6 (5.2%)	4 (8.9%)	2 (2.8%)	

IQR, interquartile range; HCV, hepatitis C virus; HBV, hepatitis B virus; HDV, hepatitis D virus; TTV, total tumor volume; PTM, post-transplant malignancies; LT-HCC, liver transplanted for HCC; LT-no-HCC, liver transplanted for other causes.

Bold values indicate p<0.05.

### Immunological Profile in LT-HCC and LT-no-HCC Patients at Baseline

At baseline, the percentages of activated cells were higher in LT-HCC than in LT-no-HCC patients (**Figure 1A**); in particular, the percentages of activated CD8^+^ T cells and memory B cells were significantly higher in LT-HCC compared to LT-no-HCC patients (%CD8^+^CD38^+^HLA-DR^+^: 10.89 (5.61-18.52) *vs* 6.59 (4.26-9.25), p=0.003; %CD19^+^CD10^-^CD21^-^CD27^+^: 10.97 (5.59-20.68) *vs* 7.60 (3.00-13.72), p=0.040) (**Figure 1A**), and a trend to significance was also observed for activated CD4^+^ T cells (%CD4^+^CD38^+^HLA-DR^+^: 7.23 (4.11-14.12) *vs* 6.21 (3.49-9.23), p=0.092) (**Figure 1A**). These results were confirmed in a logistic regression model; subjects with high percentages of activated CD8^+^, CD4^+^ T cells and B cells were at higher risk of being LT-HCC than subjects with low percentages of cells showing features of immune activation (**Table 2**).

**Table 2:** Logistic model evaluating the immunological parameters associated with HCC at baseline.

Functional phenotype*		HCC/N	OR (95%CI)**	p-value**
**%CD8^+^ activation**	Low	18/71	1	
**(CD8^+^CD38^+^HLA-DR^+^)**	High	19/31	3.9 (1.6-10.1)	**0.003**
**%CD4 activation**	Low	27/87	1	
**(CD4^+^CD38^+^HLA-DR^+^)**	High	11/17	3.8 (1.3-12)	**0.015**
**%B activated memory**	Low	24/78	1	
**(CD19^+^CD10^-^CD21^-^CD27^+^)**	High	14/23	4.0 (1.5-11.4)	**0.006**
**% CD8^+^ exhaustion**	Low	19/63	1	
**(CD8^+^PD-1^+^)**	High	17/34	2.1 (0.9-5.3)	0.096
**%CD4^+^ exhaustion**	Low	18/61	1	
**(CD4^+^PD-1^+^)**	High	19/39	2.0 (0.8-4.7)	0.122
**%B exhaustion**	Low	1/9	1	
**(CD19^+^PD-L1^+^)**	High	32/83	4.5 (0.9-45.2)	0.068
**%CD8^+^ senescence**	Low	21/75	1	
**(CD8^+^CD28^-^CD57^+^)**	High	17/22	6.0 (2.1-19.7)	**0.0007**
**%CD4^+^ senescence**	Low	22/79	1	
**(CD4^+^CD28^-^CD57^+^)**	High	16/21	6.0 (2.1-19.7)	**0.0007**
**%B senescence**	Low	17/54	1	
**(CD19^+^CD27^-^IgD^-^)**	High	13/19	3.0 (1.0-9.9)	0.054

*Categorized data on HCC to obtain optimal cut points to categorize a continuous predictor variable in a logistic regression model.

**Adjusted by age as continuous variable.

Bold values indicate p<0.05.

At baseline, no difference in the number of circulating immune cells showing markers of exhaustion was found between the two groups (%CD8^+^PD-1^+^: 9.82 (5.30-17.00) *vs* 7.54 (4.00-12.92), p=0.254; %CD4^+^PD-1^+^: 9.28 (5.66-13.50) *vs* 7.27 (4.54-10.34), p=0.222; %CD19^+^PD-L1^+^: 4.96 (3.12-9.75) *vs* 5.16 (2.18-9.00), p=0.797) (**Figure 1B**). However, in the logistic regression model, high percentage of exhausted T and B cells tended to be associated with a higher risk of being LT-HCC **(** **Table 2** **).**

LT-HCC patients had significantly higher percentage of senescent-like T and B cells compared to LT-no-HCC (%CD8^+^CD28^-^CD57^+^: 11.06 (6.24-25.16) *vs* 5.92 (3.54-10.97), p=0.006; %CD4^+^CD28^-^CD57^+^: 3.80 (1.36-14.03) *vs* 1.80 (0.48-3.41), p=0.002; %CD19^+^CD27^-^IgD^-^: 12.20 (6.28-17.67) *vs* 6.59 (4.24-12.50), p=0.019) (**Figure 1C**). These results were confirmed by the logistic model; patients with high percentages of senescent-like CD8^+^, CD4^+^ T and B cells were at higher risk of being LT-HCC (**Table 2**).

### Immune Activation and Immune Senescence Profiles at Baseline as Prognostic Markers of PTM

After a median period of follow-up of 26.8 months (10.2-42.6) following LT, 6 patients developed a PTM: 4 patients (8.9%) (3 DNMs and 1 an HCC recurrence) were in the LT-HCC group, and 2 (2.8%, both DNMs) were in the LT-no-HCC group (**Table 1**).

To evaluate the role of immune activation, exhaustion and senescence as possible markers predictive of the risk of tumor development in this setting, we analyzed these immunological parameters at baseline in samples obtained from the 6 LT-PTM patients in comparison with those of 110 LT-no-PTM patients. At baseline, the two groups did not differ by age, sex, HCV and HBV infection (**Table 1a Supplementary**).

**Figure 2:**
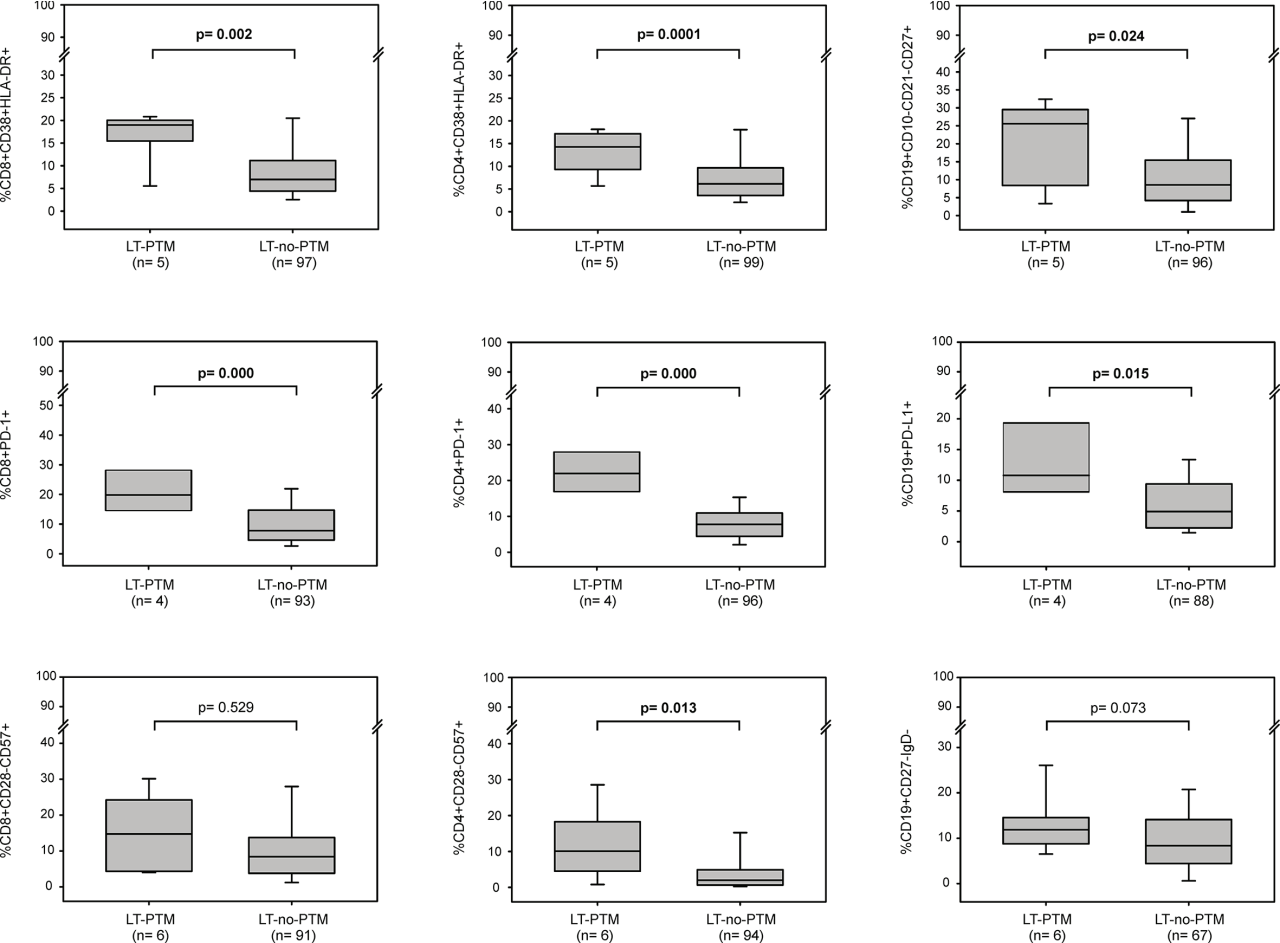
Baseline immune activation, exhaustion and senescence profiles in LT-PTM and LT-no-PTM patients. Percentages of **(A)** activated CD8^+^ (CD3^+^CD8^+^CD38^+^HLA-DR^+^), CD4^+^ (CD3^+^CD4^+^CD38^+^HLA-DR^+^) and memory B (CD19^+^CD10^-^CD21^-^CD27^+^) cells; **(B)** exhausted CD8^+^ (CD3^+^CD8^+^PD-1^+^), CD4^+^ (CD3^+^CD4^+^PD-1^+^) and B (CD19^+^PD-L1^+^) cells, **(C)** senescent-like CD8^+^ (CD3^+^CD8^+^CD28^-^CD57^+^), CD4^+^ (CD3^+^CD4^+^CD28^-^CD57^+^), and B (CD19^+^CD27^-^IgD^-^) cells in LT-PTM and LT-no-PTM patients. All p-values were adjusted by age.

The percentages of activated CD8^+^, CD4^+^ T cells and memory B cells were significantly higher in LT-PTM than in LT-no-PTM patients (%CD8^+^CD38^+^HLA-DR^+^: 19.00 (18.76-19.76) *v*s 7.14 (4.48-11.98), p=0.002; %CD4^+^CD38^+^HLA-DR^+^: 14.28 (10.53-16.84) *vs* 6.26 (3.58-9.77), p=0.0001; %CD19^+^CD10^-^CD21^-^CD27^+^: 25.57 (10.09-28.57) *vs* 8.55 (4.20-15.40), p=0.024) (**Figure 2A**). These results were confirmed in the logistic regression model (**Table 3**).

**Table 3:** Logistic model evaluating the immunological parameters associated with PTM at baseline.

Functional phenotype*		PTM/N	OR (95%CI)**	p-value**
**%CD8 activation**	Low	1/86	1	
**(CD8^+^CD38^+^HLA-DR^+^)**	High	4/16	17.5 (3.0-181.2)	**0.002**
**%CD4 activation**	Low	1/78	1	
**(CD4^+^CD38^+^HLA-DR^+^)**	High	4/26	11.2 (1.9-117.8)	**0.007**
**%B activated memory**	Low	2/86	1	
**(CD19^+^CD10^-^CD21^-^CD27^+^)**	High	3/15	10.2 (1.8-70.4)	**0.011**
**%CD8^+^ exhaustion**	Low	1/82	1	
**(CD8^+^PD-1^+^)**	High	3/15	11.7 (1.7-129.6)	**0.013**
**%CD4^+^ exhaustion**	Low	0/74	1	
**(CD4^+^PD-1^+^)**	High	4/26	25.5 (2.6-3434.9)	**0.004**
**%B exhaustion**	Low	1/64	1	
**(CD19^+^PD-L1^+^)**	High	3/28	5.2 (0.8-56)	0.090
**%CD8^+^ senescence**	Low	3/78	1	
**(CD8^+^CD28^-^CD57^+^)**	High	3/19	3.6 (0.70-19.5)	0.132
**%CD4^+^ senescence**	Low	1/70	1	
**(CD4^+^CD28^-^CD57^+^)**	High	5/30	8.4 (1.5-84.7)	**0.013**
**%B senescence**	Low	0/28	1	
**(CD19^+^CD27^-^IgD^-^)**	High	6/45	8.4 (0.9-1117.5)	0.067

*Categorized data on PTM to obtain optimal cut points to categorize a continuous predictor variable in a logistic regression model.

**Adjusted by age as continuous variable.

Bold values indicate p<0.05.

The percentage of circulating immune cells expressing markers of exhaustion was significantly higher in LT-PTM than in LT-no-PTM patients (%CD8^+^PD-1^+^: 19.81 (16.97-28.90) *vs* 7.78 (4.62-14.67), p=0.000; %CD4^+^PD-1^+^: 22.71 (17.96-27.89) *vs* 7.75 (4.47-10.85), p=0.000; %CD19^+^PD-L1^+^: 10.15 (7.82-19.75) *vs* 5.01 (2.23-9.09), p=0.015) (**Figure 2B**). These results were confirmed in the logistic regression model, which showed that high percentages of exhausted T and B cells were associated with a higher risk of being in the PTM group (**Table 3**).

The percentage of senescent-like CD4^+^ T cells and B cells tended to be higher in the LT-PTM than in LT-no-PTM patients (**Figure 2C**). This trend to significance was confirmed in the logistic regression model; high percentages of senescent-like CD4^+^ T cells and B cells were associated with a higher risk of being in the PTM group (**Table 3**).

To estimate the impact of HCC as a cause of transplantation on the development of a PTM, the LT-PTM patients were stratified according to their LT-HCC and LT-no-HCC status and compared to overall LT-no-PTM patients. Several observations, taken with caution given the low number of PTM, were possible. At baseline, percentages of activated and exhausted CD8^+^, CD4^+^ T cells and B cells were higher in LT-PTM patients compared to LT-no-PTM patients in both groups (**Figure 2 supplementary**). Notably, within the LT-HCC group the percentages of senescent-like CD8^+^ and CD4^+^ T cells were significantly higher in LT-PTM patients than in LT-no-PTM patients (**Figure 2 supplementary**).

### Immune Activation and Immune Senescence Profiles at Follow-Up in LT-PTM and mLT-no-PTM Patients

To further investigate overtime the association between immune activation and immune senescent profiles and onset of PTM, the immunological parameters were evaluated and compared between LT-PTM patients and a matched group of 26 mLT-no-PTM (see *Materials and Methods* and **Table 1b Supplementary**) at follow-up (i.e. closest to the tumor onset in LT-PTM patients and with the same median time interval for mLT-no-PTM patients) (**Table 2a Supplementary**).

At follow-up, the percentages of activated and exhausted CD8^+^, CD4^+^ T cells and B cells were significantly higher in LT-PTM patients compared to mLT-no-PTM (**Table 2a Supplementary**). Regarding senescence, levels of senescent-like B cells were significantly higher in LT-PTM compared to mLT-no-PTM (%CD19^+^CD27^-^IgD^-^: 19.39 (18.56-20.95) *vs* 10.08 (5.33-12.83), p=0.002), while no difference was observed in senescent-like CD8^+^ and CD4^+^ T cells (**Table 2a Supplementary**). These results were confirmed by logistic regression model (**Table 2b Supplementary**).

### Circulating Levels of PAMPs and DAMPs

**Figure 3:**
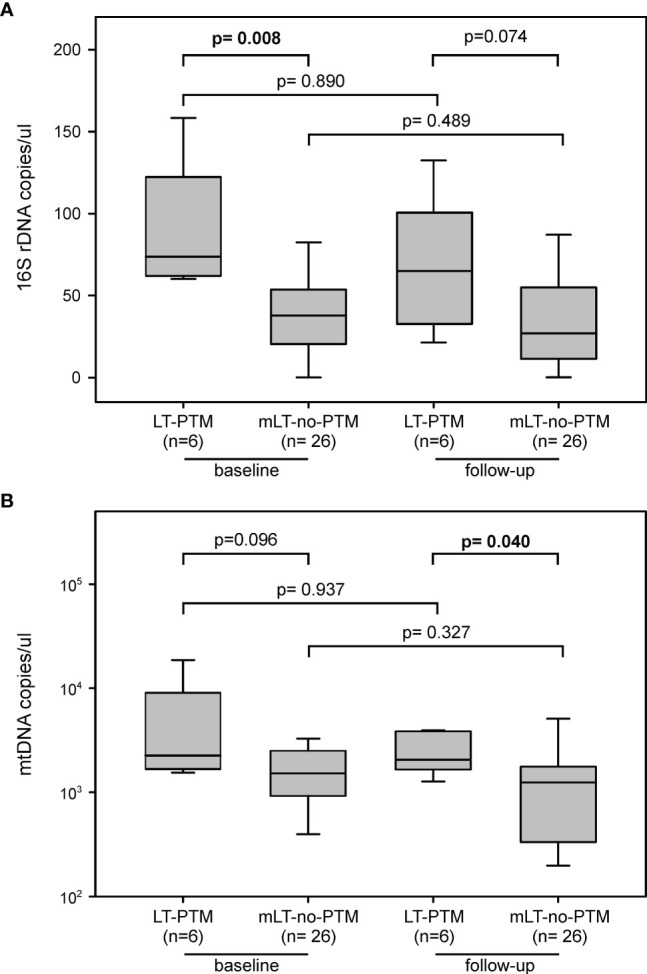
Circulating markers of microbial translocation between LT-PTM and mLT-no-PTM at baseline and follow-up. Circulating levels of **(A)** PAMPs (16S rDNA) and **(B)** DAMPs (mtDNA) in LT-PTM *versus* mLT-no-PTM patients at baseline and follow-up.

At baseline, levels of 16S rDNA were significantly higher in LT-PTM compared to mLT-no-PTM (73.69 (61.38-120.94) *vs* 37.79 (17.99-54.06) copies/µl, p=0.008); since no difference occurred in the two groups during follow-up, this level still remained higher at follow-up (64.95 (29.51-106.15) *vs* 26.99 (11.27-58.74) copies/µl, p= 0.074) (**Figure 3A**). Similarly, at baseline, mtDNA levels tended to be higher in LT-PTM compared to mLT-no-PTM (3369 (2169-9570) *vs* 1822 (985-3984) copies/µl, p=0.096), but they become significantly higher at follow-up (3075 (2187-6244) *vs* 1668 (447-2424) copies/µl, p= 0.040) (**Figure 3B**).

### Telomere Length

At baseline, relative telomere lengths (RTL) on PBMC were longer, although not statistically significant, in LT-PTM than in mLT-no-PTM patients (RTL: 1.46 (1.03-1.63) *vs* 1.08 (0.80-1.63), p=0.391). Notably, at follow-up, the levels did not differ between the two groups (RTL: 0.79 (0.73-0.80) *vs* 0.77 (0.75-0.83), p=0.873), thus indicating that LT-PTM patients tended to have a higher telomere erosion during the same follow-up interval (Δ_FU-baseline_: -0.66 (-0.75; -0.29) *vs* -0.09 (-0.48; -0.04), p=0.091).

## Discussion

Liver transplantation represents the best treatment choice for patients with acute or chronic liver failure and for a fraction of those with HCC (6); however, after successful liver transplantation, *de novo* malignancies (DNMs) and HCC recurrence are the most common causes of long-term morbidity and death (7, 8, 25). The need of identifying biological markers predictive of the risk to develop post-transplant malignancies is therefore clinically important. To the best of our knowledge, this is the first study assessing the possible role of immune activation, exhaustion, and senescence profiles as biomarkers potentially useful to identify liver transplanted patients at higher risk to develop post-transplant tumors.

The gut-liver axis is widely implicated in the pathogenesis of liver diseases and most of the gut bacterial communities have the capacity to colonize multiple tumor milieu (26, 27). Detrimental inflammatory responses after solid organ transplantation are initiated when immune cells bind, through the Toll-like receptor, PAMPs and DAMPs released during transplant-associated processes (i.e. ischemia/reperfusion injury, surgical trauma, and recipient conditioning) and/or the constant exposure to triggering insults in the liver (as chronic viral hepatitis infection or alcohol use) (14). Translocation of PAMPs and DAMPs into circulation may lead to the abnormal activation of the immune system, which is considered a critical step for post-transplant tumorigenesis (9, 28). However, very few data are available on the role of aberrant immune activation in the complex process of cancerogenesis occurring in liver transplanted patients. A previous study suggested no difference in the expression of activated CD8^+^CD38^+^ T cells between patients who developed malignancies and non-cancer patients (29), but in a Danish cohort of solid organ transplant recipients, older age and elevated levels of C-reactive protein, a circulating marker of inflammation, correlated with a high risk for cancer development (30). In this study, levels of 16S rDNA and mtDNA, markers of PAMPs and DAMPs respectively, were higher at baseline in LT-PTM than in LT-no-PTM patients and remained higher at tumor onset. Consistently, immune activation identified by high numbers of CD38^+^HLA-DR^+^ T cells and CD10^-^CD21^-^CD27^+^ B cells, was associated with a higher risk of developing a post-transplant malignancy, regardless of the cause of liver transplantation. Overall, these data suggest that the persistent immune activated status, likely induced by circulating levels of microbial translocation markers after transplantation, *itself* represents an important risk factor for tumor onset.

Aging is a well-known risk factor for tumor development. The exhaustion and senescence of T cells are two dysfunctional states in chronic infections and cancers (31, 32). Differently from exhausted T lymphocytes, senescent cells may retain their cytotoxic potential and ability to secrete high levels of inflammatory cytokines, contributing to the SASP phenotype that plays crucial roles in promoting cancer (33–35). Senescent T cells accumulate in the peripheral blood of patients with solid tumours and represent a potential prognostic biomarker (32, 33). Here, we found that patients who developed a PTM tended to have at baseline higher levels of immune senescent-like cells than no-PTM patients; in particular, within the cohort of patients transplanted for HCC, the numbers of circulating senescent-like CD4^+^ and CD8^+^ T cells were significantly higher, thus suggesting that immune senescence at baseline may be an additional predictive marker in patients transplanted for HCC. Our results concerning the telomere length tend to support this possibility. Telomeres are repetitive DNA sequences located at the ends of the chromosomes and intact telomeres are essential for genomic integrity and the maintenance of the cellular proliferative potential (36). Inflammation and telomere shortening are hallmarks of the early stages of chronic liver disease and play a pivotal role in the early onset of neoplasia (37, 38). In a previous study, we found that low telomere length is associated with a higher risk of tumor onset (17). In the transplantation field, shorter telomere length in peripheral blood leukocytes was associated with a significantly worse clinical response in patients receiving immune checkpoint inhibitor therapy across different malignancies and the calculated relative telomere length differences defined as “delta telomere length” was found to be an independent predictor of overall survival (39). In our study, liver transplanted patients who developed a tumor presented a longer telomere length at baseline, but tended to undergo to a higher telomere erosion between the baseline and the tumor onset compared to LT-no-PTM patients during the same follow-up period.

In conclusion, our findings suggest that circulating biomarkers of immune activation and exhaustion may usefully predict the risk of tumor development in liver transplanted patients, regardless of the cause of transplantation. At time of transplantation, LT-HCC carried an immune activated status, most likely derived from the primary tumor, which was not solved by liver transplantation and persisted long after that, leading to a higher accumulation of immune senescent cells and a progressive telomere erosion. These abnormalities could contribute to an enhanced and accelerated promotion of tumorigenesis in liver transplanted patients resulting in an earlier onset of post-transplant cancers, as compared to patients transplanted for other causes. Confirmation of these findings in a larger prospective series of liver transplanted patients may provide the rationale for the exploitation of immune activation, exhaustion and senescence profiles as clinically useful markers to be included in the follow up laboratory routine to identify patients at higher risk to develop post-transplant tumors.

## Data Availability Statement

The raw data supporting the conclusions of this article will be made available by the authors, without undue reservation.

## Ethics Statement

The studies involving human participants were reviewed and approved by Ethical Committee of Padova University Hospital (Prot. 4231/AO/17). The patients/participants provided their written informed consent to participate in this study.

## Author Contributions

MRP, SS, MT, PP, DS, PB and ADR, designed the study. MRP, ER, FC, SG, performed the investigations. SS, DS, UC, PB, provided samples and reagents. PDB performed the statistical analysis. MRP wrote the manuscript. SS, MT, SG, PDB, PP, DS, UC, RD, PB and ADR supervised the project, reviewed and editing the manuscript. All authors contributed to the article and approved the submitted version.

## Funding

This work was supported by Ricerca Corrente IRCCS Centro di Riferimento Oncologico, Aviano: Italian Association for Research on Cancer; Grant number: AIRC IG No. 19112; and “Pathogenesis of EBV-driven post-transplant malignancies” granted by Department of Surgery, Oncology and Gastroenterology (DiSCOG), University of Padova, Italy (Grant no. BIRD181981/18, PI ADR).

## Conflict of Interest

The authors declare that the research was conducted in the absence of any commercial or financial relationships that could be construed as a potential conflict of interest.

## Publisher’s Note

All claims expressed in this article are solely those of the authors and do not necessarily represent those of their affiliated organizations, or those of the publisher, the editors and the reviewers. Any product that may be evaluated in this article, or claim that may be made by its manufacturer, is not guaranteed or endorsed by the publisher.
