# Supplementary material for: Immune Activation, Exhaustion and Senescence Profiles as Possible Predictors of Cancer in Liver Transplanted Patients
Source: Front Oncol. 2022 Jun 13;12:899170. doi: 10.3389/fonc.2022.899170 (PMC9235349; doi:10.3389/fonc.2022.899170)
Supplement: Supplementary file 1 [file DataSheet_1.docx]

**Table 1a supplementary.** Baseline characteristics of LT-PTM and LT-no-PTM patients

|  |  | **Total**  **patients** | **LT-PTM**  **patients** | **LT-no-PTM patients** | **p-value** |
| --- | --- | --- | --- | --- | --- |
|  | N | 116 | 6 | 110 |  |
| **Age (years)** | Median (IQR) | 56.0 (46.8-62.0) | 62.0 (60.5-62.0) | 56.0 (46.3-62.0) | 0.150 |
| **Sex** | F | 36 (31.0%) | 2 (33.3%) | 34 (30.9%) | 0.901 |
|  | M | 80 (69.0%) | 4 (66.7%) | 76 (69.1%) |  |
| **HCV** | No | 98 (84.5%) | 5 (83.3%) | 93 (84.5%) | 0.936 |
|  | Yes | 18 (15.5%) | 1 (16.7%) | 17 (15.5%) |  |
| **HBV** | No | 90 (77.6%) | 5 (83.3%) | 85 (77.3%) | 0.636 |
|  | Yes | 13 (11.2%) | 1 (16.7%) | 12 (10.9%) |  |
|  | Yes + HDV | 13 (11.2%) | 0 (0.0%) | 13 (11.8%) |  |
| **LT-HCC** | No | 71 (61.2%) | 2 (33.3%) | 69 (62.7%) | 0.190 |
|  | Yes | 45 (38.8%) | 4 (66.7%) | 41 (37.3%) |  |

LT-PTM: Liver Transplanted with Post-Transplanted Malignancies; LT-no-PTM: Liver Transplanted without Post-Transplanted Malignancies; HDV: hepatitis D virus; LT-HCC: liver transplanted for HCC.

**Table 1b supplementary.** Baseline characteristics of LT-PTM and mLT-no-PTM patients.

|  |  | **Total**  **patients** | **LT-PTM**  **patients** | **mLT-no-PTM patients** | **p-value** |
| --- | --- | --- | --- | --- | --- |
|  | N | 32 | 6 | 26 |  |
| **Age (years)** | Median (IQR) | 61.0 (49.8-63.3) | 62.0 (60.5-62.0) | 59.0 (49.3-63.8) | 0.884 |
| **Sex** | F | 11 (34.4%) | 2 (33.3%) | 9 (34.6%) | 0.952 |
|  | M | 21 (65.6%) | 4 (66.7%) | 17 (65.4%) |  |
| **HCV** | No | 28 (87.5%) | 5 (83.3%) | 23 (88.5%) | 0.732 |
|  | Yes | 4 (12.5%) | 1 (16.7%) | 3 (11.5%) |  |
| **HBV** | No | 28 (87.5%) | 5 (83.3%) | 23 (88.5%) | 0.415 |
|  | Yes | 2 (6.2%) | 1 (16.7%) | 1 (3.8%) |  |
|  | Yes + HDV | 2 (6.2%) | 0 (0.0%) | 2 (7.7%) |  |
| **LT-HCC** | No | 13 (40.6%) | 2 (33.3%) | 11 (42.3%) | 0.687 |
|  | Yes | 19 (59.4%) | 4 (66.7%) | 15 (57.7%) |  |
| **Time of follow-up** | Median (IQR) | 12.04 (5.62-24.19) | 15.38 (7.46-23.49) | 9.00 (5.76-23.78) | 0.629 |

LT-PTM: Liver Transplanted with Post-Transplanted Malignancies; LT-no-PTM: Liver Transplanted without Post-Transplanted Malignancies; HDV: hepatitis D virus; LT-HCC: liver transplanted for HCC.

**Table 2a supplementary.** Immunological profile in LT-PTM patients and mLT-no-PTM at follow-up.

| **Functional phenotype** | **LT-PTM patients**  Median (IQR) | **mLT-no-PTM patients**  Median (IQR) | **p-value** |
| --- | --- | --- | --- |
| **%CD8 activation (CD8+CD38+HLA-DR+)** | 17.55 (14.10-19.20) | 2.78 (2.00-3.82) | **0.0001** |
| **%CD4 activation (CD4+CD38+HLA-DR+)** | 11.79 (11.06-11.80) | 4.04 (2.44-5.85) | **0.008** |
| **%B activated memory (CD19+CD10-CD21-D27+)** | 5.63 (3.47-7.43) | 2.75 (1.40-4.75) | **0.034** |
| **% CD8 exhaustion**  **(CD8+PD-1+)** | 34.24 (31.76-39.52) | 16.39 (8.49-22.74) | **<0.0001** |
| **%CD4 exhaustion**  **(CD4+PD-1+)** | 24.78 (21.52-32.75) | 17.16 (11.79-25.38) | **0.031** |
| **%B exhaustion**  **(CD19+PD-L1+)** | 6.02 (5.88-8.10) | 2.38 (1.34-6.21) | 0.078 |
| **%CD8 senescence**  **(CD8+CD28-CD57+)** | 29.63 (15.26-37.40) | 23.02 (15.12-32.35) | 0.825 |
| **%CD4 senescence**  **(CD4+CD28-CD57+)** | 15.00 (9.58-21.99) | 9.31 (5.71-14.46) | 0.489 |
| **%B senescence**  **(CD19+CD27-IgD-)** | 19.39 (18.56-20.95) | 10.08 (5.33-12.83) | **0.002** |

Bold values indicate p<0.05

**Table 2b supplementary.** Logistic Model evaluating the immunological parameters in predicting PTM at follow-up

| **Functional phenotype*** |  | **PTM/N** | **OR (95%CI)** | **p-value** |
| --- | --- | --- | --- | --- |
| **%CD8 activation** | Low | 0/26 | 1 |  |
| **(CD8+CD38+HLA-DR+)** | High | 5/5 | 583 (24.8-188343) | **<0.0001** |
| **%CD4 activation** | Low | 1/25 | 1 |  |
| **(CD4+CD38+HLA-DR+)** | High | 4/6 | 29.4 (3.7-410.4) | **0.001** |
| **%B activated memory** | Low | 2/22 | 1 |  |
| **(CD19+CD10-CD21-D27+)** | High | 3/9 | 4.4 (0.7-32.2) | 0.112 |
| **% CD8 exhaustion** | Low | 1/25 | 1 |  |
| **(CD8+PD-1+)** | High | 4/6 | 29.4 (3.7-410.4) | **0.001** |
| **%CD4 exhaustion** | Low | 0/11 | 1 |  |
| **(CD4+PD-1+)** | High | 5/20 | 8.2 (0.8-1112.2) | 0.084 |
| **%B exhaustion** | Low | 1/20 | 1 |  |
| **(CD19+PD-L1+)** | High | 4/11 | 7.8 (1.2-88.1) | **0.032** |
| **%CD8 senescence** | Low | 2/18 | 1 |  |
| **(CD8+CD28-CD57+)** | High | 3/13 | 2.2 (0.4-15.3) | 0.386 |
| **%CD4 senescence** | Low | 2/21 | 1 |  |
| **(CD4+CD28-CD57+)** | High | 3/10 | 3.6 (0.6-26.1) | 0.162 |
| **%B senescence** | Low | 1/22 | 1 |  |
| **(CD19+CD27-IgD-)** | High | 4/8 | 14.3 (2-172.5) | **0.007** |

*Categorized data on PTM to obtain optimal cut points to categorize a continuous predictor variable in a logistic regression model. Bold values indicate p<0.05

**Figure 1 supplementary. Gating strategy for flow cytometry analysis.** (A) Cells were first gated for lymphocytes (FSC *versus* SSC). (B) Lymphocyte gate was further analysed for uptake of the Live/Dead Fixable Near-IR Dead to determine live *versus* dead cells. (C) The expression of CD3+CD4+ or CD3+CD8+ was determined on live lymphocytes. (D)Within CD3+CD4+, CD38 and HLA-DR were used to identify activated cells (CD38+HLA-DR+), PD-1 to mark exhausted cells (PD-1+), and CD57 and CD28 to define senescent-like cells (CD28‐CD57+). (E) Within CD3+CD8+, CD38 and HLA-DR were used to identify activated cells (CD38+HLA-DR+), PD-1 to mark exhausted cells (PD-1+), and CD57 and CD28 to define senescent-like cells (CD28‐CD57+).

(F) The expression of CD19+ B cells was determined on live lymphocytes. (G) Whitin the CD19+, CD10, CD27, CD21 markers were used to identify activated memory (CD10-CD27+CD21-), CD27, IgD to define senescent-like cells (IgD-CD27-), and (H) PD-L1 to select exhausted cells (PD-L1+).


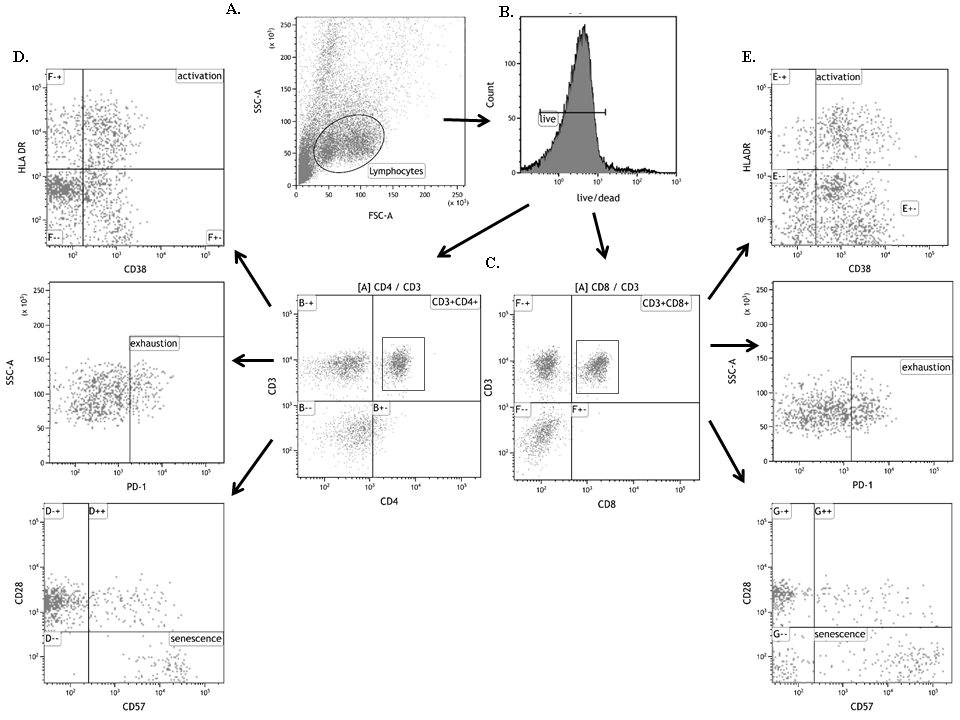


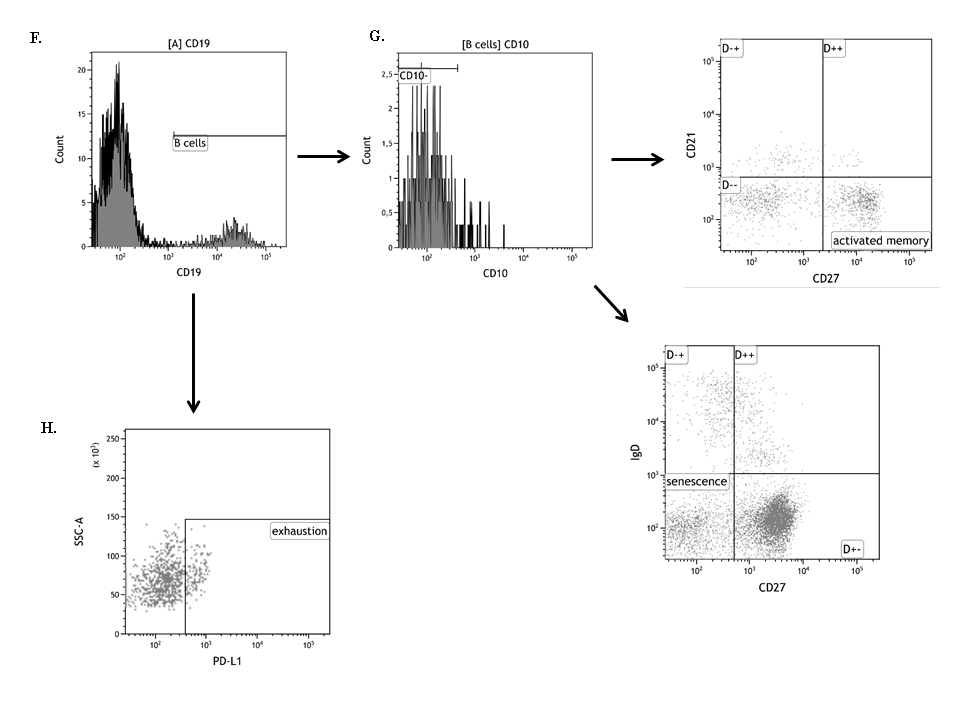


**Figure 2 supplementary. Baseline immune senescence in PTM stratified according to LT-HCC and LT-no-HCC and no-PTM**. Percentages of (A) activated CD8 (CD3+CD8+CD38+HLA-DR+), CD4 (CD3+CD4+CD38+HLA-DR+) and memory B (CD19+CD10-CD21-CD27+) cells; (B) exhausted CD8 (CD3+CD8+PD-1+), CD4 (CD3+CD4+PD-1+) and B (CD19+PD-L1+) cells; (C) senescent-like CD8 (CD3+CD8+CD28-CD57+), CD4 (CD3+CD4+CD28-CD57+), and B (CD19+CD27-IgD-) cells in PTM in LT-HCC and LT-no-HCC patients and no-PTM patients at baseline. All p-values were adjusted by age.

**
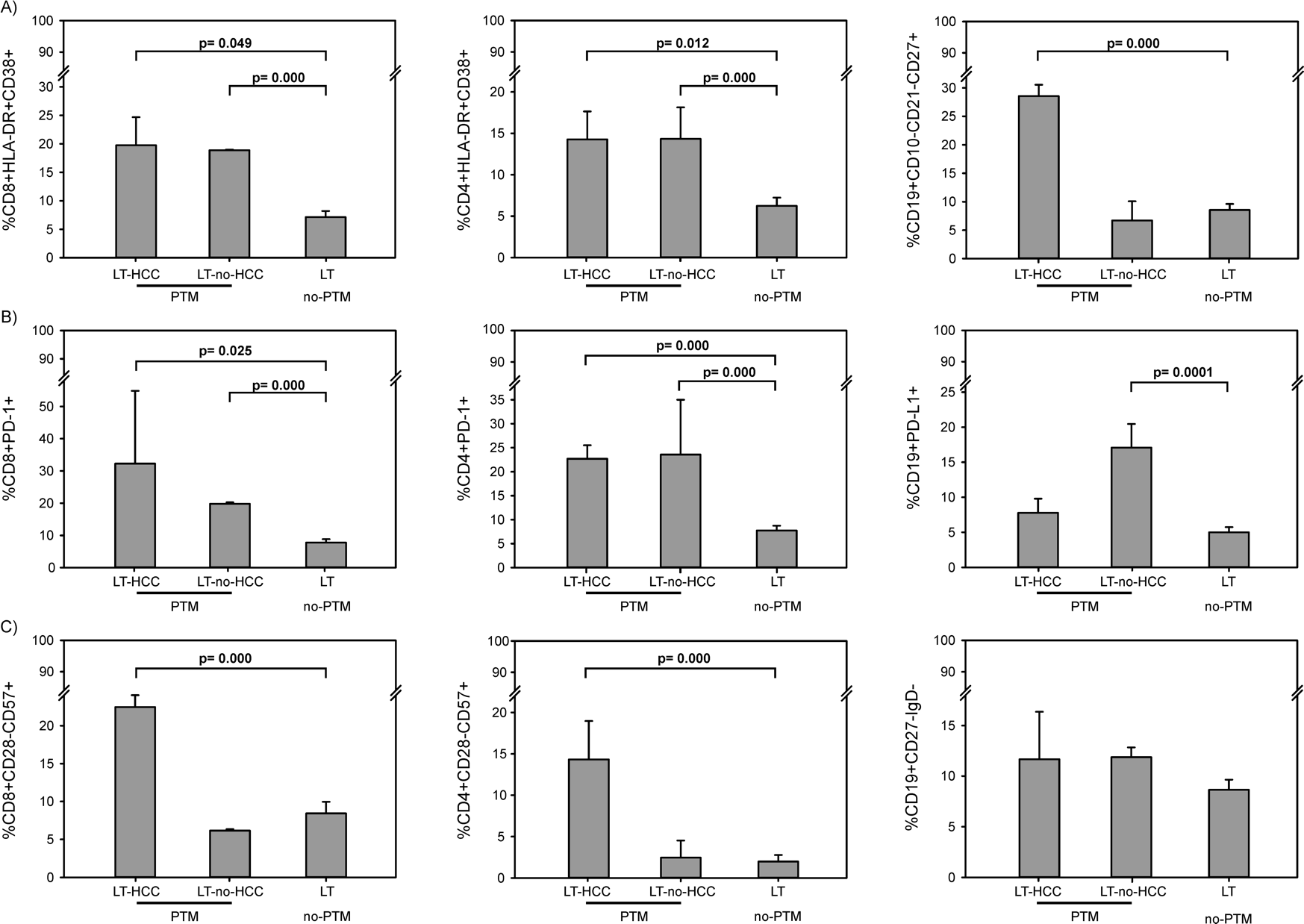
**
